# Effects of Ultraviolet Light Irradiation on Silk Fibroin Films Prepared under Different Conditions

**DOI:** 10.3390/biom11010070

**Published:** 2021-01-07

**Authors:** Sora Lee, Soo Hyun Kim, You-Young Jo, Wan-Taek Ju, Hyun-Bok Kim, HaeYong Kweon

**Affiliations:** Sericultural and Apicultural Materials Division, National Institute of Agricultural Sciences, RDA, Wanju-gun 55365, Korea; ysr357@gmail.com (S.L.); hyunsk0513@gmail.com (S.H.K.); yyjo@korea.kr (Y.-Y.J.); wantaek@korea.kr (W.-T.J.); hyunbok@korea.kr (H.-B.K.)

**Keywords:** silk fibroin film, ultraviolet aging, photo-oxidation, photo-degradation

## Abstract

Silk fibroin (SF)-based materials are exposed to both natural and artificial ultraviolet (UV) light during preparation or administration. However, the effects of UV irradiation on SF films prepared under different conditions have not yet been described in detail. In this study, four SF films with different molecular weight (MW) distribution were fabricated using SF solutions, which were prepared by dissolving degummed SF for 0.5–24 h. We observed UV (365 nm) irradiation on SF films induced the increase of yellowness and absorbance at 310 nm of SF films, indicating the formation of new photo-products and di-tyrosine bonds by photo-oxidation. Due to di-tyrosine cross-links between SF chains, UV-irradiated SF films were not fully dissociated in urea solution. In addition to formation of new products, UV reduced the crystallinity of SF films by breaking hydrogen bonds of β-sheet conformation. Unlike the UV-induced decomposition of physical interactions, UV did not affect the covalent bonds (i.e., peptide bonds). Through these experiments, we could expect that SF with higher MW was more susceptible and SF with lower MW was more resistant to UV-induced photo-oxidation and photo-degradation. These results provide useful information about UV-induced aging of SF-based materials under natural sunlight and UV irradiating conditions.

## 1. Introduction

Silk fibroin (SF) is one of well-known natural protein materials obtained from silkworms. The primary structure of SF is highly unique and composed of repetitive glycine-alanine-glycine-alanine-glycine-serine (GAGAGS) [1]. These repeating sequences form the crystalline region with β-sheet conformation maintained by various molecular interactions such as hydrogen bonds, van der Waals interaction, and hydrophobic interaction. The crystalline regions contribute to the strength of SF. In contrast, amorphous regions of relatively hydrophilic blocks composed of random/α-helix conformations contribute to the elasticity of SF. 

Because of the high crystallinity of the degummed SF, SF has been generally regarded as water-insoluble polymer. Instead of pure water, several chaotropic solvents [2] have been used for solubilization and application of SF in a variety of forms such as films, sponges, hydrogels, or coating materials [3]. During the solubilization process of degummed SF, the structure of SF can be affected by various conditions such as the type of solvent, dissolution time, and temperature, resulting in differences in physical and chemical features of SF. For example, different solvent systems that have different ionic forces can affect the molecular weight (MW) and structure of SF [4]. Wang and colleagues compared the MW of SF solutions dissolved in 9.3 M LiBr and Ajisawa’s reagent (CaCl_2_/H_2_O/EtOH (molar ratio 1/8/2)) by using Rouse-like rheological behavior, which enables the determination of molecular weight of SF [2]. M_W_ of LiBr-dissolved SF was 179 kDa, whereas M_W_ of Ajisawa’s reagent-dissolved SF was 128 kDa, indicating that LiBr solvent system was milder. In fact, Yamada et al. also found that Ajisawa’s reagent caused degradation of the heavy chain of SF [5]. In addition, the fast protein liquid chromatogram of Ajisawa’s reagent-dissolved SF showed a strong peak indicating 450 kDa and weak shoulder peak indicating 150 kDa. The peak of 150 kDa increased with the increase of dissolution time. Moreover, peak shift from 150 kDa to 110 kDa and new peak formation indicating 16 kDa were observed when dissolution time was 180 min [6]. Such a different MW distribution resulted in different particle size, rheological properties, and degree of transparency. Meanwhile, according to the literature, the gelling property of SFs triggered by ultrasonic treatment was poor in Ajisawa’s reagent system compared to LiBr system, although the similar MW and distribution of SFs were acquired by the control of dissolution time [7]. It can be expected that these two solvent systems might affect not only MW distribution of SF but also its structure of hydrophobic segments. Hence, the dissolution conditions must be set carefully considering the target application fields.

In addition to various dissolution conditions, there are various external parameters that can affect the physicochemical properties of SF, such as heat, oxygen, volatile organic compounds, light, or absolute humidity [8]. Among them, ultraviolet (UV) light is a highly important parameter because during preparation and administration, SF-based fibers, medical devices, and additives for foods and cosmetics are generally exposed to natural or artificial UV light. Especially, UV sterilization of SF-based biomaterials is the required procedure before implantation [9]. Therefore, understanding the physicochemical stabilities of SF-based material under UV light is essential for a wide range of applications.

For several decades, many researchers have tried to understand the UV-induced photo-aging process of silk fabrics, solutions, and films. The accumulated results have demonstrated that SF fiber is susceptible to UV-induced changes in color and brightness [10,11] because SF molecules contain numerous aromatic amino acids such as tyrosine (5.1%), tryptophan (0.1%), and phenylalanine (0.4%) [8]. In addition, it is revealed that photo-destruction of the ordered structures by UV irradiation makes silk fibers more brittle than those that are nontreated [12]. To reduce UV-induced weakening effect on mechanical properties, researchers fabricated silk fibers coated with TiO_2_ as UV-absorbers and confirmed more stable tensile properties [13]. Sionkowska and colleagues also found that thin films from a mixture of chitosan and silk fibroin were affected by UV light with a wavelength of 254 nm, showing the decrease of surface roughness that was detected by atomic force microscopy [14]. 

Although photo-induced oxidation and degradation are highly important reactions for using SF-based materials, to the best of our knowledge, the effect of dissolution time and the resultant different MW distribution on the resistance of SF-based materials against the photo-induced reaction have not been investigated in detail. Therefore, in this study, four types of SF solutions were prepared by dissolving degummed SF fibers for different times (0.5–24 h). Subsequently, SF films were fabricated and irradiated by UV light. In addition, spectroscopic and structural properties of UV-irradiated SF films were investigated.

## 2. Materials and Methods 

### 2.1. Preparation of SF Aqueous Solutions

Degumming of *Bombyx mori* cocoons was performed twice with 0.5% o.w.f. (on the weight of fiber) of sodium oleate (C_18_H_33_NaO_2_, Sigma Aldrich, MO, USA) and 0.3% o.w.f. of solution sodium carbonate (Na_2_CO_3_, Sigma Aldrich) with a bath ratio of 1:50 at 100 °C for 1 h. The silk fibers were rinsed in warm water and air-dried at 50 °C. The degummed silk was dissolved in Ajisawa’s reagent solution containing calcium chloride (CaCl_2_, Showa, Japan), distilled water, and ethanol with molar ratio of 1:8:2 at 87 °C for 0.5 h, 2 h, 8 h, and 24 h. In 0.5 h-dissolution group, some undissolved fibers remained. However, no residual fibers were observed in the other groups. Next, SF solutions were dialyzed using a cellulose acetate tube (MWCO: 12–14 kDa) for 3 days. To remove insoluble components, filtration processes were performed before and after dialysis. The final concentration of aqueous SF solution was measured by weighing the remaining solid after drying at 130 °C. Six milliliters of 1.6 wt.% regenerated SF solutions were poured into 6-well plates and dried in fume hoods to prepare regenerated SF films.

### 2.2. Sodium Dodecyl Sulfate-Polyacrylamide Gel Electrophoresis (SDS-PAGE) and Silver Staining

Protein concentration of each SF solution was measured using Bradford assay (Bio-Rad laboratories, Hercules, CA, USA) and the solutions were mixed with 2X Laemmle buffer (Bio-Rad) and 2.5% β-mercaptoethanol. Denatured SF samples at 95 °C for 2 min were loaded into the lanes of acrylamide gel (Bio-Rad) and electrophoresis was performed. Next, the gel was stained using a silver staining kit (Invitrogen, Carlsbad, CA, USA) according to the manufacturer’s guide. Using the analyze line graph tool of the image processing software (ImageJ), the gray value profile was created along each lane of the gel image. The representative profile of three replicates was selected and shown herein.

### 2.3. UV Irradiation on SF Films

SF films were irradiated in air at room temperature using a UV lamp (LF215LM, UVITEC, Cambridge, UK) which emits light at a wavelength of 365 nm. The average intensity of radiation was 2.37 ± 2.12 mW/cm^2^ at 5 cm from the light source. The dose of radiation during 1 h exposure was 8538 ± 453 mJ/cm^2^. The intensity of the incident light was measured using an UV light meter (YK-35UV, Lutron, Taiwan). Irradiation experiments were carried out on a well-plate and the location of films were rotated every 15 min for the uniform exposure of light.

### 2.4. Color Measurement

Color change of SF films was measured using a spectrophotometer (Color i7, X-Rite, Grand Rapids, MI, USA). A white standard color plate (L = 94.27, a = 0.13, and b = 2.80) was used as a background and a standard for color measurements. Hunter color values (L: lightness; a: redness; b: yellowness) were averaged from more than 10 measurements from each sample. The total color differences (∆E) were calculated as follows:

∆E = [(∆L)^2^ + (∆a)^2^ + (∆b)^2^ ]^0.5^ (1)

where ∆L, ∆a, and ∆b are the difference between each color values of film specimen and the standard.

### 2.5. UV–Vis Absorbance and Fluorescence Spectroscopy

The UV–vis absorption spectra of SF film before and after UV-irradiation were recorded with a Multiskan GO plate reader (Thermo Scientific, Waltham, MA, USA) in the range of 270 to 360 nm. Films were fixed inside of the cuvette cell. Absorbance of SF molecules released from SF films during immersion in 6 M urea solution at 10 mg/mL was also collected. The fluorescence intensity (excitation: 360/40 nm; emission: 460/40 nm) of the SF molecules in 6 M urea solution was measured by microplate reader (HT-Synergy, Bio-Tek, Winooski, VT, USA). For film and solution samples, the measured values of empty cuvette and the same volume of 6 M urea solution were used for blank subtraction, respectively.

### 2.6. Attenuated Total Reflection Fourier Transform Infrared (ATR-FTIR) Spectroscopy

ATR-FTIR experiments were carried out and the spectra of silk films were recorded with a Fourier transform spectrometer (S100, Perkin Elmer, Waltham, MA, USA) in the range of 1000 to 4000 cm^−1^. Thirty-two scans were averaged with resolution 4 cm^−1^ and the experiments were repeated more than 6 times. Amide I and III crystallinity index were determined by calculating the absorbance intensity ratios of 1620 cm^−1^/1652 cm^−1^ for amide I and 1264 cm^−1^/1230 cm^−1^ for amide III crystallinity index.

### 2.7. Assay of 2,4,6-Trinitrobenzene Sulfonic Acid (TNBS)

To determine the relative content of primary amines in SF film, TNBS assay was performed according to the established protocol with minor modification [15]. SF films (2.5 mg) were mixed with 250 μL of 200 mM sodium phosphate buffer (pH 8.2) and 250 μL of 0.5% of TNBS solution. The reaction mixture was heated at 40 °C for 4 h in a shaking incubator. To stop the reaction, 250 μL of 6 N HCl was added into the mixture. Next, the mixture was autoclaved at 120 °C for 30 min to dissolve any insoluble matter. The absorbance of 100 μL of mixture was read at 340 nm using a microplate reader (Multiskan GO) and all values were subtracted by the value of blank solution prepared with same procedure without SF film.

## 3. Results and Discussion

### 3.1. Characterization of Molecular Weight Distribution of SF Dissolved for Different Time 

**Figure 1:**
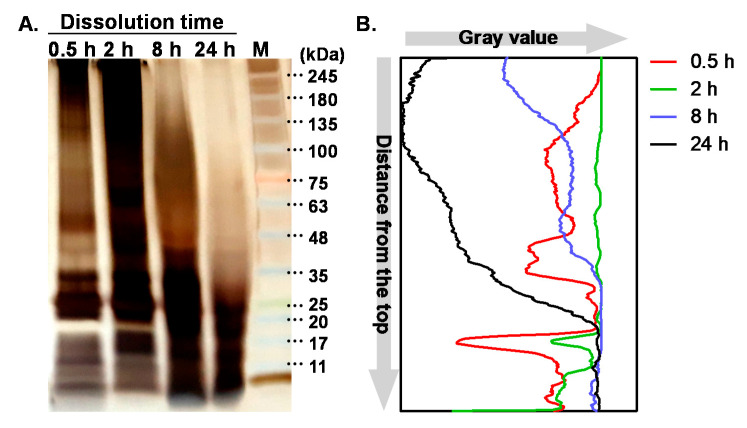
SDS-PAGE results of four silk fibroin (SF) solutions. (**A**) Silver-stained gel image showing the difference of SFs in molecular weight distribution. The lane on the right side indicates the molecular weight marker. The other four lanes represent SF with increasing dissolution time, from left to right. (**B**) The gray value profiles of protein bands on the SDS-PAGE gel. The set of intensity values is taken from each lane, from the top to the bottom, and plotted in the chart.

Although Ajisawa’s reagent has been used for dissolving the degummed SF fibers with high utility, it can make damages in the intrinsic structure of SF, resulting in molecular scissions [5,6]. As expected, SDS-PAGE results shown in Figure 1A indicate 0.5 h and 2 h SF groups that were dissolved for a relatively short time showed strong band intensity at high MW region (>100 kDa). Unlike the low band intensity of 0.5 h SF group between 100 and 35 kDa, the band intensity for the 2 h SF group was high between the same range, indicating the wide distribution of MW for the 2 h SF group. In the case of the 8 h SF group, band intensity was gradually increased in the range from 180 kDa to the bottom of the gel. For the 24 h SF group, which was dissolved for the longest time, a strong band was shown only at the lower MW region (<35 kDa). Molecular distributions of SF groups dissolved for different times were also observed in the gray value profile of each lane of SDS-PAGE gel (Figure 1B). Considering SF had a heavy chain of 391 kDa and a light chain of 26 kDa [16], we could expect the bands’ upper region of 245 kDa and near region of 25 kDa were contributed by the heavy and light chain of SF, respectively. Therefore, the bands from 245 kDa to 25 kDa and below 25 kDa were thought to be the degraded heavy and light chain, respectively. According to the results showing the weak band intensities of the 8 and 24 h groups, we could conclude that dissolution for longer than 8 h could almost critically degrade the heavy chains of SF fibers. 

### 3.2. Effect of UV Irradiation on Color Change of SF Films 

**Figure 2:**
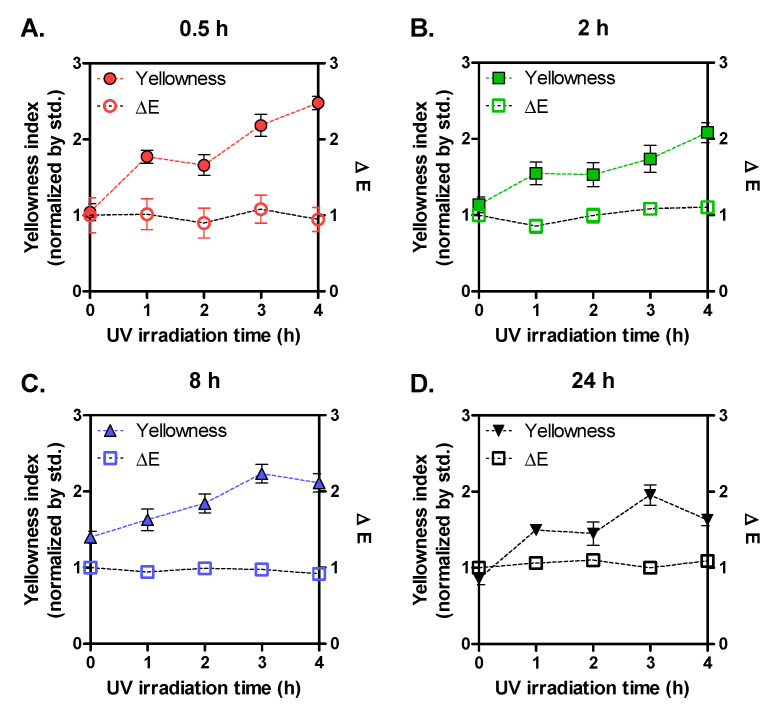
Yellowness and total color differences (ΔE) of UV-irradiated SF films (1–4 h) prepared with four SF solutions that were dissolved for different times (**A**: 0.5 h; **B**: 2 h; **C**: 8 h; **D**: 24 h) (n > 10; mean ± SEM).

The difference in MW distribution of SF can generally affect molecular structure and physicochemical properties. Given the SDS-PAGE results, we hypothesized that SF groups dissolved for different times would show different susceptibility to UV irradiation. To confirm this, dose-dependent color changes were evaluated. Total color differences (∆E) calculated from values of Hunter L (lightness), a (redness), and b (yellowness) for the SF films were plotted with yellowness index (b) as a function of UV irradiation time. As depicted in Figure 2, ∆E was not affected by UV irradiation time, regardless of dissolution time. In contrast, yellowness index tended to increase with UV irradiation. Especially, the yellowing effect on the 0.5 SF group was stronger than other groups, showing more than a 2-fold increase of yellowness index after 4 h of UV irradiation. In contrast, the yellowness indexes of the other groups were lower than the 0.5 h group even after 4 h of irradiation. Yellowing is considered as one of the most important photo-oxidation markers in proteins. The development of yellow color of SF films might be because aromatic amino acids produced new products containing chromophores under UV light [17]. Utilizing a quasiproteomic approach, Dyer and colleagues identified chromophoric photo-products derived from tyrosine and tryptophan, and proposed the photo-oxidation pathways [18]. Therefore, these color measurement results indicate the MW of SF can affect the susceptibility to UV-induced production of chromophores. We also expected that UV irradiation can be an ecosustainable technique for modifying color properties of SF film without using chemical reagents. Similarly, Sagnella and colleagues modified the color properties of silk fiber and silk fibroin by doping RhoB in the artificial diet during silkworm raising [19]. With the increase of feeding time of RhoB-added diet from 0 to 72 h, chromaticity (C) increased and hue angle (*h*°) decreased, indicating the color change from between bluish-green and yellow components to an almost red-purple component. 

### 3.3. Effect of UV Irradiation on UV–Vis and Fluorescence Spectroscopic Properties of SF Films

**Figure 3:**
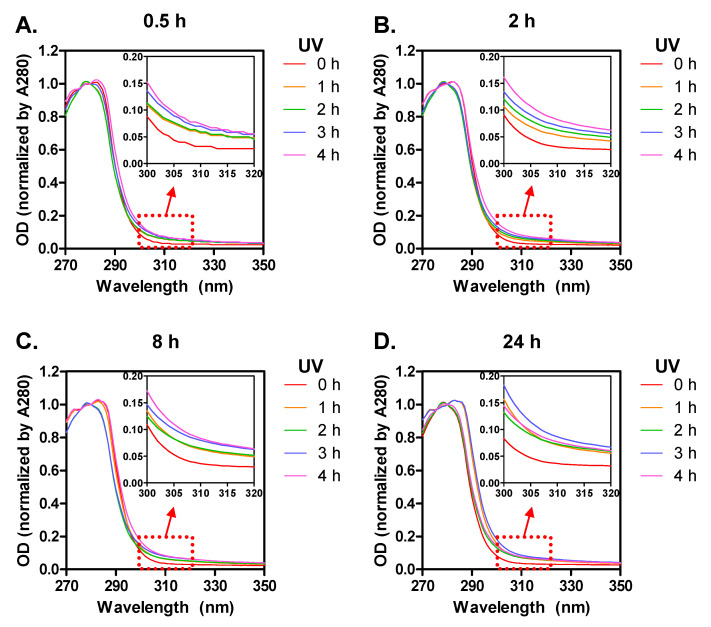
Absorbance spectra of UV-irradiated SF films (1–4 h) prepared with four SF solutions that were dissolved for different times (**A**: 0.5 h; **B**: 2 h; **C**: 8 h; **D**: 24 h).

Changes of UV–vis absorbance spectra of SF films can detect the formation of new photo-products. As shown in Figure 3A–D, the main peak around 280 nm is attributed to aromatic amino acids that are present in SF molecular chains such as tyrosine (5.1%), phenylalanine (0.4%), and tryptophan (0.1%) [1,8]. After UV irradiation, light absorbance at 310 nm tended to increase for all SF groups. These results are probably due to the formation of new photo-products by UV irradiation such as new cross-links and/or an oxidized form of aromatic amino acids. Similarly, Sionkowska and colleagues also observed the changes in absorbance of SF solutions at around 300–310 nm and they explained that 3,4-dihydroxylphenylalanine (DOPA) might be formed by photo-oxidation reaction between phenylalanine and tyrosine, affecting the absorbance property [1]. 

**Figure 4:**
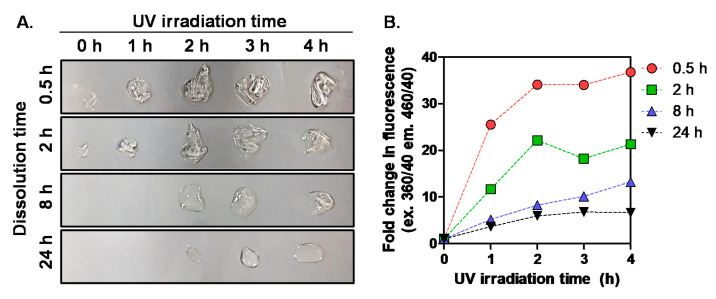
Morphologies of the remaining four SF films (**A**) and fluorescence intensity of the released SF molecules from SF films (**B**) after immersion of films in 6 M urea solution for 24 h (ex. 360/40; em. 460/40) (n = 3; mean ± SD; 1-fold: 0 h UV irradiation).

To know the presence of newly formed cross-links between SF chains, we immersed SF films in 6 M urea solution at 10 mg/mL and wait for 24 h. Because it is well known that concentrated urea solution can easily break hydrogen bonds in SF, we expected SF films would be dissolved. In fact, intact SF films were almost dissolved, remaining few solid-state components, as shown in Figure 4A. However, with the increase of UV irradiation time, larger amounts of undissolved and swollen films were observed for all SF groups, indicating SF chains were photo-cross-linked by chemical bonds instead of hydrogen bonds. The chemical bonds formed by UV irradiation are thought to be di-tyrosine bonds. To confirm the formation of di-tyrosine bonds in released SF molecules, fluorescence intensity at 440–480 nm was measured at the excitation wavelength of 320–380 nm. According to the literature, for UV irradiated insulin, the progressive formation of a peak around 405 nm was detected when samples were excited at 320 nm due to di-tyrosine bonds [20]. Similarly, for all SF samples, the fluorescence was increased with the increase of UV irradiation time, indicating the formation of di-tyrosine bonds (Figure 4B). These di-tyrosine bonds might be formed upon tyrosyl radical isomerization and enolization reaction [21]. It is especially worth nothing that SF groups with higher MW showed a higher increase of fluorescence upon increase of UV irradiation time. This fluorescence profile is similar to the color measurement result in Figure 2A–D and film morphologies shown in Figure 4A. Therefore, we can conclude the MW of SF can influence the susceptibility to UV in terms of formation of new photo-products. These MW effects might be related to the content of aromatic amino acids because longer heat treatment time could damage more aromatic residues during dissolution of degummed SF fibers.

### 3.4. Effect of UV Irradiation on Structural Stability of SF Films

**Figure 5:**
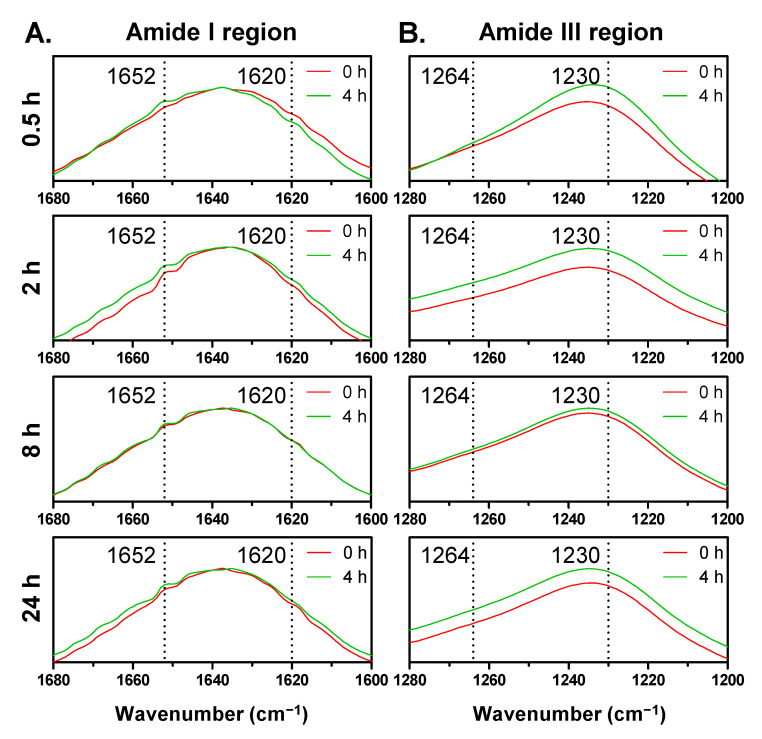
FT-IR spectra of SF films nonirradiated and irradiated with UV light for 4 h; (**A**) amide I region (1680–1600 cm^−1^), (**B**) amide III region (1280–1200 cm^−1^).

**Figure 6:**
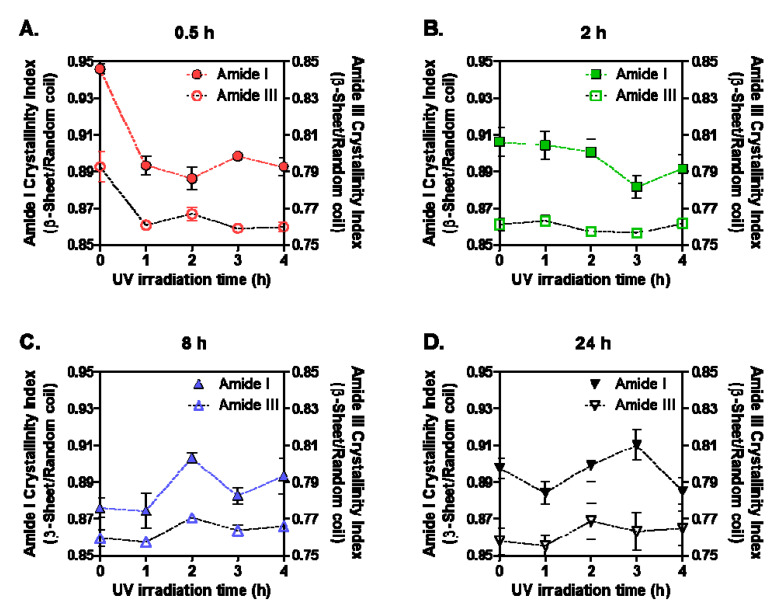
Amide I and III crystallinity index calculated from FT-IR spectra of UV-irradiated SF films prepared with four SF solutions that were dissolved for different times (**A**: 0.5 h; **B**: 2 h; **C**: 8 h; **D**: 24 h) (n > 6; mean ± SEM).

In addition to formation of new chemical bonds by UV, the high energy of UV can induce photo-degradation of hydrogen bonds and disulfide bonds. Therefore, we evaluated by FT-IR the structural stability of four different SF films after exposure to UV. FT-IR is very useful tool for understanding the chemical and physical structure of silk proteins. In the literature, a semiquantitative approach was chosen by calculating the relative intensities of amide bands to know the extent of SF degradation [8]. The ratios of two amide band intensities that indicate random/α-helix and β-sheet conformations have been used as one of degradation markers by comparing the crystallinity. Therefore, we acquired FT-IR spectra and calculated the ratio of 1620 cm^−1^ (β-sheet) to 1652 cm^−1^ (random coil) for amide I crystallinity index (CI) and 1264 cm^−1^ (β-sheet) to 1230 cm^−1^ (random coil) for amide III CI. The FT-IR spectra of nonirradiated and 4 h irradiated SF films showed different absorbance intensities at the specific wavenumbers (cm^−1^), which indicated β-sheet and random coil structures (Figure 5). In the case of SF films prepared by a shorter dissolution time (0.5 h and 2 h SF groups), the absorbance values at 1652 and 1230 cm^−1^ indicated random coil structure increased after 4 h UV irradiation, whereas the values at 1620 and 1264 cm^−1^ indicated β-sheet structure decreased or increased slightly. On the other hand, in the case of the 8 h and 24 h SF groups, there were no significant changes in the absorbance values, thus indicating both structures. Especially, both values at 1264 and 1230 cm^−1^ of the 24 h SF group increased similarly after UV irradiation, meaning there were less compositional changes of β-sheet and random coil that occurred with UV irradiation. Moreover, as shown in Figure 6, we could compare the calculated CI values upon the irradiation time. Like the tendency for formation of photo-products, the 0.5 SF group showed a strong decrease of both amide I and III CI. The destruction of crystalline structure was thought to be affected by breakage of hydrogen bonds involved in β-sheet conformation (Figure 6A). In addition, this result can explain the previously reported weakened mechanical properties of aged silk fibers under UV exposure [12,13]. Meanwhile, the 2 h SF group showed a slight increase of amide I CI by 1 h UV irradiation and a gradual decrease after 1 h (Figure 6B). The newly formed di-tyrosine cross-links could dominantly contribute to an increase of CI. However, when the dose of UV light was accumulated, the decomposition effects of UV might reduce the crystallinity by breaking hydrogen bonds, like the 0.5 SF group. For the 8 h and 24 SF groups, the CI values were not dramatically changed (Figure 6C,D). We could expect that SF chains with higher MW were extensively affected by UV energy and the physical interactions were easily broken because a larger number of hydrogen bonds were involved in long SF chains than in shorter chains.

**Figure 7:**
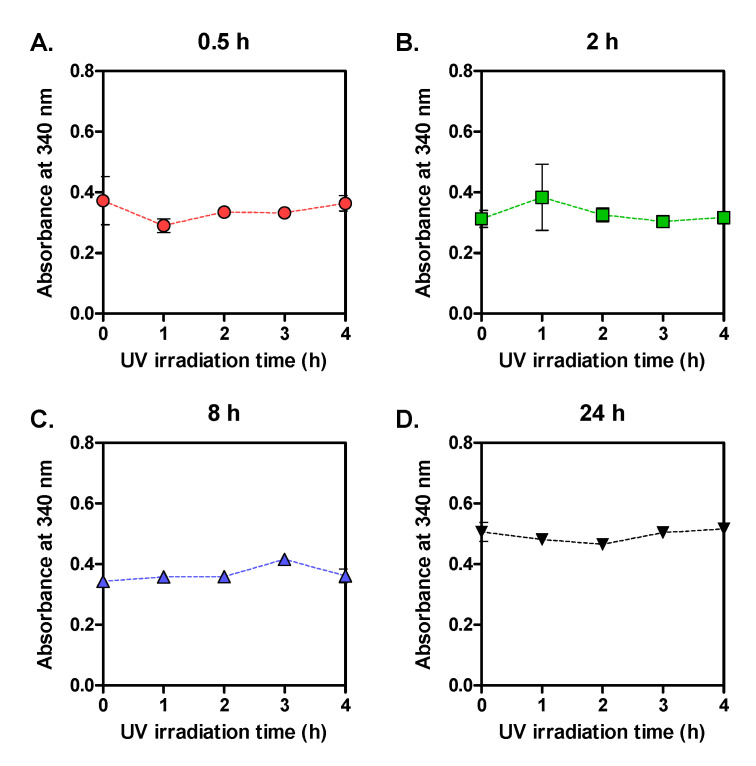
Absorbance at 340 nm obtained by 2,4,6-trinitrobenzene sulfonic acid (TNBS) assay with UV-irradiated SF films prepared with four SF solutions. The SFs were dissolved for different times (**A**: 0.5 h; **B**: 2 h; **C**: 8 h; **D**: 24 h) (n = 3; mean ± SD).

From the TNBS assay results, we could not observe any remarkable differences in the content of ε-amine group or newly formed N-terminal amine groups (Figure 7). These results indicate that UV did not induce chain scission and decrease MW for all SF groups. Because peptide bonds are covalent, unlike the crystallinity that is maintained by physical molecular interactions, stronger energy may be needed to break the peptide bonds. Considering that several other studies reported the molecular degradation by UV [22], light with higher energy or longer irradiation time than the light used for our study might induce chain scissions and decrease the MW of SF.

## 4. Conclusions

In this study, investigation on the effect of UV irradiation on the physicochemical properties of SF films prepared under different conditions was carried out. UV irradiation on SF films not only formed of new bonds by photo-oxidation, but also damaged crystallinity of SF by photo-degradation. These UV-induced photo-reactions were confirmed by measuring color, UV–vis absorbance spectra, fluorescence, or FT-IR spectra. In addition, we could observe that SF with higher MW was more susceptible to these UV irradiation effects. To explain the exact mechanism about MW effect on photo-resistance of SF films, further studies in the molecular levels may be required. In conclusion, we expect that our study would provide useful information for preparing and applying SF-based materials under natural sunlight or UV irradiating conditions.
